# Upgrading of bio‐separation and bioanalysis using synthetic polymers: Molecularly imprinted polymers (MIPs), cryogels, stimuli‐responsive polymers

**DOI:** 10.1002/elsc.202100106

**Published:** 2022-02-21

**Authors:** Sevgi Aslıyüce, Neslihan Idil, Bo Mattiasson

**Affiliations:** ^1^ Department of Chemistry Biochemistry Division Hacettepe University Ankara Turkey; ^2^ Department of Biology Biotechnology Division Hacettepe University Ankara Turkey; ^3^ Department of Biotechnology Lund University Lund Sweden; ^4^ Indienz AB Annebergs Gård, Billeberga Lund Sweden

**Keywords:** cryogels, molecular imprinting, separation, smart polymers, synthetic polymers, two‐phase systems

## Abstract

Bio‐separation plays a crucial role in many areas. Different polymers are suitable for bio‐separation and are useful for applications in applications in both science and technology. Besides biopolymers, there are a broad spectrum of synthetic polymers with tailor‐made properties. The synthetic polymers are characterized by their charges, solubility, hydrophilicity/hydrophobicity, sensitivity to environmental conditions and stability. Furthermore, ongoing developments are of great interest on biodegradable polymers for the treatment of diseases. Smart polymers have gained great attention due to their unique characteristics especially emphasizing simultaneously changing their chemical and physical property upon exposure to changes in environmental conditions. In this review, methodologies applied in bio‐separation using synthetic polymers are discussed and efficient candidates are focused for the construction of synthetic polymers.

## 1 INTRODUCTION

Natural polymers have been used in both bio‐separation [1] and bioanalysis [2]. The inborn properties of these polymers are that they are sensitive to denaturation and also to degradation by enzymes [3]. These factors are leading to a need for more robust polymers, and that is why synthetic polymers are evaluated as replacements to the natural polymers. An advantage of synthetic polymers is that one can modify the properties of the polymer by using different monomers in the polymerization process. In this context, new polymers formed by changing some of the physical properties and chemical functions appear as promising tools in different application areas. Synthetic polymers are potential materials to provide high selectivity, simplicity of process, low cost and moderate working conditions, which are very important for bio‐separation, thanks to their wide variety of synthesis conditions, polymerization patterns and functionalization [4].

Bio‐separation relies on the interaction between target molecule/cell and support material [5]. Polymers can be prepared depending on the application. Examples of these polymeric materials are microspheres [6], membranes [7], nanostructures [8], monoliths [3] and cryogels [7]

Separation has a crucial role in biotechnological research and various fundamental studies have addressed the conventional precipitation methods. Therefore, several chromatographic steps are needed to obtain successful separation efficiencies. Affinity or biorecognition ligands are effectively preferred to gain selective interaction between target and support material. Due to the rapid developments in the polymer industry, smart polymers are sensitive to environmental factors such as temperature, pH, ionic strength, humidity, electric current, magnetic field, changing their function in line with the direction determined by a change in these materials. In the meantime, smart polymers take the place as the next generation of bio‐separation tools [9].

Among separation technologies, aqueous two‐phase separation has been introduced as an well‐established alternative over conventional ones such as distillation, precipitation, filtration, crystallization, due to providing mild and biocompatible conditions, high water capacity, low interfacial stress, and convenience in combined systems [10].

Molecular recognition‐based approaches have gained considerable interest from many researchers and bring a new dimension to analytical bio‐separation. Molecularly imprinted polymers (MIPs) have very similar identities compared to those of natural counterparts such as antibodies, enzymes, or receptors and carry well‐established biorecognition features [7]. In recent years, due to the advances, initiatives and contributions in the field of preparation of MIPs took place. At the moment, they are developed commercially opening new possibilities for research and it is probably that they will find widespread applications.

Use of synthetic polymers for the production of MIPS which has led to production of ‘plastibodies’ (instead of antibodies). These synthetic ‘plastibodies’ have shown very good selectivity and sensitivity equal to what good antibodies have or even better ‐ combined with high stability and robustness towards microbial enzymes [4, 5].

MIPs are produced by mixing a target molecule which one wants to give an imprint in the polymer after it has been polymerized. Thus, the target molecule is mixed with the monomers so that the target molecules are present in the polymer after polymerization. Then, a cavity selective will be left after the target molecule has been removed which is capable of re‐bind the target molecule [4].

The number of approaches to the application of synthetic polymers is growing. One example within the field of application is biomedicine. In one concept, biodegradable polymeric materials have been widely used for the treatment of bone‐based diseases and dental issues. Besides, the recent trend of their applications in drug delivery and orthopedic implants, biodegradable polymers are receiving more attention [11]. Synthetic polymers find application in diagnostics and many attempts were made in clinical trials [12, 13]. They have a crucial role in early diagnostics, monitoring and even prevention of diseases including cancer [14].

Separation technologies such as chromatography‐based methods, centrifugation, electrophoretic tools, filtration, flow cytometry, magnetism‐based techniques and sensing devices are used by commercial platforms [3, 6].

The continuous advancements of new technologies in the field of bio‐separation including development in bioprocessing equipment and reagents are helping to expand the economics area of bio‐separation applications. In line with the increasing interest of the science community in biosimilars,chemicals are designed to look similar to earlier studied molecules [15].

Applications of the MIPs expand within the bio‐separation as well as in bio‐based analysis, for example, via biosensors with MIPs for example, attention in the lab‐on‐a‐chip [16].

This review is the focused on three different areas where synthetic polymers are used; cryogels which are gels built from synthetic polymers and have porosity that is far more pronounced than what is seen in from natural polymers. In the present review, a brief overview is given by describing the fundamentals and advantages of (1) synthetic polymers considering bio separation and analytical systems. Applications of synthetic polymers such as smart polymers, (2) cryogels, composite materials and (3) MIPs, generated for bio‐separation are broadly explained. Lastly, challenges and future perspectives are discussed.

## 2 POLYMERS UTILIZED IN BIO‐SEPARATION

In biochemical methods, extraction, purification and separation have been preferred. For these methods, gelatin, chitin, chitosan, cellulose, starch, and pectin were applied and they offer the opportunity to enhance modifications [17]. By this way, desired features such as biodegradability, biocompatibility, and stability can be obtained by modifications. The advantages of natural polymers can be listed as easy‐obtainability at low price and availability in large quantities, non‐toxic effects, offering modification and high biocompatibility [18].

Besides, chromatography is a frequently used separation method, when isolating small molecules. Normally organic solvents were mixed with aqueous solutions of homogenized biomass. Use of organic solvents is connected with environmental problems, so extraction methods are less attractive if environmental concerns are relevant [19].

The development and presentation by Per‐Åke Albertsson of aqueous two‐phase systems represented a very important step in bio‐separation [20].

Two polymer‐solutions are mixed to create aqueous two‐phase separation systems. The basic concept is mixing a solution of two dissolved polymers in water one could see that it was feasible to get a emulsion between the two phases due to low compatibility between the different polymers. It became obvious that some molecules were partitioned to one of the phases while others could be recovered from the other phase [21].

Apart from polymer/polymer couple, polymer/salt, ionic and/or non‐ionic surfactants, inorganic salt/short‐chain alcohols also can be applied for the generation of these systems. It was possible to change the partitioning between the phases using different agents. In literature, it has been reported that cells, enzymes and proteins were efficiently separated using these systems [21].

Initially, most often dextran and poly ethylene glycol (PEG) are used. We even cultivated microorganisms in such a system and could harvest viable cells while the extracted low‐molecularly products that were recovered from the other phase ref. Furthermore, mammalian cells could be treated in the two‐phase system without losing viability [22]. The two‐phase systems were useful for separation of proteins, cells and cell organelles. Albertsson et.al. isolated thylakoids from the leaves of spinach [23]. The overview of the two‐phase systems applied for the separation of different targets is given in Table 1. A positive feature of the aqueous two‐phase system is that it is easy to scale up.

**TABLE 1:** The overview of the two‐phase systems applied for the separation of different targets

Type of	Material	Target molecule	Principle	Ref.
Polymer/polymer	Charged polymer: polyethyleneimine (PEI) and an uncharged polymer: dextran (DEX), hydroxyethylcellulose (HEC) and polyethylene glycol (PEG).	Organic acid resulted from fermentation.	pH sensitive system was constructed by PEI titrated with H_2_SO_4_ and H_3_PO_4._ Low pH resulted in higher phase ratio with smaller bottom (PEIrich) phase.	[27]
Polymer/polymer	PEI, HEC	Cultivation of Lactococcus lactis and lactic acid production	PEI/HEC systems was able to make lag phase longer.	[28]
Polymer/salt	PEG/phosphates	Clavulanic acid	pH had a negligible effect on the yield and high temperature resulted in low yield	[29]
Polymer/salt	PEG/Na_2_SO_4_	Butyric acid from the fermentation broth	Butyric acid, acetic acid, and butanol were found in PEG‐rich top phase. Iodine was used to precipitate PEG and then, butyric acid was separated.	[30]
Ionic liquids and deep eutectic solvents	[Hmim]Br/(NH)_2_SO_4_	Succinic acid	Alcohols/salts‐based systems were investigated and it was found that pH does not affect the effectiveness of the proposed system. The extraction of succinic acid from top was lower in the presence of ionic liquids.	[31]
Alcohol/salt	*t*‐Butanol and different salts	Succinic acid	The extraction of succinic acid using 1‐propanol and different salts showed that high distribution coefficients were obtained with NaCl and (NH_4_)_2_SO_4_. The lower equilibrium pH of the bottom salt‐rich phase leads to high obtainability.	[31]

Abbreviation: DEX, dextran; HEC, hydroxyethyl cellulose; [Hmim]Br, 1‐hexy‐3‐methylimidazolium bromide; PEG, Polyethylene glycol; PEI, polyethyleneimine.

Using these two‐phase systems, it would be possible to apply the technology when developing immunoassays in aqueous solutions. A major challenge concerning immunoassays when dealing with soluble antibodies and aggregates of antibodies which have captured antigens was to separate native antibodies from antibodies‐antigens complexes. Partition in aqueous two‐phase systems turned out to be a success. In order to amplify the partition pattern, one could modify some of the reagents. The polymers in the two‐phase systems are to some extent soluble in 'each other' phases. That leads to losses of polymers when the phases are separated and the partitioned substances have been harvested [24].

Some polymers are sensitive to environmental conditions. Some of these are gathered under the term stimuli‐responsive polymers (sometimes even 'smart polymers'). Temperature sensitive polymers are known to change conformation whereby their properties undergo a pronounced transition of their surface properties. An example is that in case that the polymer is exposed to higher temperature, the hydrophobic properties are more pronounced which results in much lower water solubility [25].

Stimuli‐responsible polymers are used in chromatography [26]. As an example, Sepharose beads were used for affinity capturing of lactate dehydrogenase. Affinity ligands were immobilized on Sepharose, for capturing the enzyme. The Sepharose beads were also exposed to a temperature sensitive polymer which was adsorbed on the chromatographic material. Upon exposure to the raised temperature, the stimuli‐responsive polymer underwent conformational changes and thereby displacing the captured enzyme that was then eluted with high yield.

## 3 SYNTHETIC POLYMERS

Various polymers with a wide range of physical, chemical and biological properties can be synthesized to meet the requirements of different applications. Most of the synthetic polymers have backbones composed of carbon‐carbon bonds. Elements such as oxygen, sulphur or nitrogen can be added near the backbone to produce heteropolymers. An example of inorganic polymers is polycyloxane with a silicon‐oxygen backbone [32].

Poly(meth)acrylates are frequently used in bio separation as hydrogels/cryogels [33] and micro/nanoparticles [34]. Methacrylates having different functional groups were commonly applied. With the advantages of these functional groups, molecular imprinting technique, state of art technology, can also be applied for obtaining selectivity. Polymethyl methacrylate (PMMA) is synthesized by crosslinking (meth)acrylate monomers. Poly(2‐hydroxyethyl methacrylate) (PHEMA) was one of the first hydrogels to be synthesized [35]. Both PMMA and PHEMA have high biocompatibility and hydrophilic properties due to their free hydroxyl groups. Thus, they were preferred to use in the synthesis of spongy and porous materials, PMMA and PHEMA based support matrices have high water retention capacity. Poly(N‐isopropylacrylamide) (PNIPAAm) has unique physicochemical properties: for example, PNIPAAm dissolves in water below the low critical solution temperature (LCST) of 32°C while forming a gel at *T* > 32°C. The reason is the formation of strong hydrogen bonds between the polymer and water molecules. Generally, poly(ethylene oxide) (PEO) and poly(ethylene glycol) (PEG) are non‐degradable polyethers. PEO is synthesized from ethylene oxide monomers. PEO has a hydroxyl group [36]. PEG has a lower molecular weight than PEO. PEG is soluble in water. High molecular weight PEGs can be prepared as hydrogels. PEG hydrogels composed of cross‐linked polymer chains are prone to aqueous hydrolysis, so they are biodegradable. Poly(lactic acid) (PLA), poly(glycolic acid) (PGA) and poly(lactic‐co‐glycolic acid) (PLGA) are polyesters of biodegradable α‐hydroxy acids. Biodegradable polymers are used in biomedical fields such as drug delivery systems and scaffolds [37] rather than bio separation.

The physical properties of polymers result from interactions between polymer chains. These interactions are hydrogen bonds and dipole‐dipole forces. These bonds form the primary structure of the polymer. The bonds formed with crosslinkers change the properties of polymers. The chain length of the polymer changes its molecular weight. Polymers containing aromatic rings are more polarized and these polymers have strong intermolecular forces. Hydrogen bonds between amides in polyamides help to stabilize them [38].

In recent years, R&D in life science and biotechnology has introduced special requirements for bio separation. With the increase in recombinant DNA technology and cell culture research, lower cost product purification and high recovery rates are required without losing its bioactivity. By the initiation of advances in precision medicine, selective capture of biomarkers is crucial for early diagnosis of disease. Studies using different synthetic polymers have been conducted in the last few years to find biomarkers by enriching the potential target from complex bio‐samples [39].

### 3.1 Stimuli‐responsive polymers (smart polymers)

Smart polymer systems undergo fast and reversible changes from hydrophilic to hydrophobic microstructure. Such microscopic changes which are triggered by small changes in the microenvironment such as temperature, pH, ionic strength, or concentration of specific substances like sugar [9].

Synthetic polymers used for bio‐separation and/or bioanalysis in characterized by using stimuli‐responsive polymers. Since the surface properties of the polymer is drastically changed when an environmental parameter is changed. Thus, when surfaces utilized for bio‐separation and bioanalysis are covered with a stimuli‐responsive polymer. (The transition temperature range for a specific polymer is narrow. However, there is a broad diversity between different polymers and the transition temperature for different polymers may vary markedly [9]. An example that illustrates the sharp transitions at changed temperatures is valid for the sharp transitions. The polymer is usually named as PNIPAm [40].

Temperature sensitive polymers exhibit swelling and shrinkage behaviour depending on their formation above and below a certain temperature. These polymers are produced for the immobilization of molecules and arrangement of cell sheets. Besides, they find application in the fields of controlled drug delivery, tissue engineering and bio‐separation devices [14]. These polymers can be generally created by adding acidic or basic functional groups to the polymer backbone. In aqueous medium, protons are given or received in response to changes in suitable pH and ionic strength [5].

pH sensitive smart polymers are functional polymers that can exchange their own configurations and properties according to pH changes. They have a hydrophobic structure obtained by the functional groups. Acidic (e.g., carboxylic or sulfonic acids) or basic (e.g., ammonium salts) groups are found in all pH sensitive polymers. Numerous polymers of ionizable groups are known as polyelectrolytes. Their solubility also changes according to pH variations showing solubility at high and low pH values called as polyanionic and polycationic polymers, respectively. One of the most used pH sensitive smart polymers contains polyacrylic acid [41]. The overview of the stimuli responsive polymers applied for the separation of different targets is given in Table 2.

**TABLE 2:** The overview of the stimuli responsive polymers applied for the separation of different targets

Polymer	Responsiveness	Target	Separation method	Ref.
PVDF/PMMA‐g‐PEO	Temperature	Bovine serum albumin	Affinity separation	[43]
PNIPAM‐TP	pH and temperature	O‐GlcNAc proteins	Affinity precipitation	[44]
PNIPAm	Temperature	Ciprofloxacin	Molecular imprinting technology	[45]
Fe_3_O_4_/CMCS/PAAPBA	pH	Glycoproteins	Boronate affinity	[46]
P(AAc‐*co*‐AAm)	pH	Thrombin	Microfludic separation	[47]
PNIPAm‐MAH	Temperature	Ni(II)	Molecular imprinting technology	[33]
p(NIPAm‐MAH)	Temperature	IgG	Molecular imprinting technology	[48]
P(HEMA)/gelatine	pH	Doxorubicin	Molecular imprinting technology	[49]

Abbreviations: Fe_3_O_4_/CMCS/PAAPBA, Fe_3_O_4_/carboxymethylated chitosan/poly(3‐acrylaminophenylboronic acid); IgG, immunglobulin G; MAH, *N*‐methacryloyl‐l‐histidine, poly(N‐isopropylacrylamide‐N methacryloyl‐(l)‐histidine; O‐GlcNAc, O‐linked β‐N‐acetylglucosamine; P(AAc‐*co*‐AAm); pNIPAm, poly(N‐isopropylacrylamide); pNIPAM‐ TP, poly(*N*‐isopropylacrylamide‐*co*‐methyl acrylate) triarylphosphine, poly(acrylamide‐*co*‐acrylic acid; PVDF/PMMA‐*g*‐PEO, poly(vinylidene difluoride)/poly(methyl methacrylate)‐g‐ poly(ethylene oxide).

pH stimuli polymers have been applied in drug and gene delivery systems, and glucose sensors in terms of their ability to make a change in vital organs or related diseases [14, 42] .

### 3.2 Superporous chromatographic materials: Cryogels

Chromatographic material is often characterized by being porous and having small pores for chromatographic separation. Porous polymers have pores in the range of 50 nm to 1 μm and these porosity figures were the standard materials especially when dealing with proteins and small biomolecules. However, when dealing with particulate materials clogging of the chromatographic material caused problems. The standard chromatographic materials were used when affinity chromatography was developed, but it still had the limitations of small pores.

Cryogels are gels that are formed in low temperatures, often at −12 to −18°C. Initially, the gels were formed on acrylics and the polymerization took place under frozen conditions. The monomers and catalysts to facilitate the polymerization procedure were then enriched in the interstitial space between the ice‐crystals formed [50]. The ice‐crystals are growing until meet another ice‐crystal, and that leads to formation of a network of ice‐crystals. The study on cryogels was by V. I. Lozinsky was later studied in our labs in order to evaluate the possibilities to use cryogels with its large pores within biotechnology [51]. Polymerization at low temperatures took several hours, 12–18 h. When the cryogels were taken to room temperature, the ice‐crystals melted and the water was removed. Monomers that were not included in the polymer are washed out and so are also done for oligomers which now also some natural polymers are included in the polymer network. When the ice‐crystals melt it leaves a network of polymer filaments with large pores. The resultant cryogels have pores with the diameters changed between 50 nm and 100 μm. The preparation of cryogels were usually catalysed by free radicals crosslinking co‐polymerization of vinyl monomers and crosslinking monomers [7].

By manipulating the temperature for the polymerization, one could control the crystal formation such that at very low temperature, more crystals were formed. The size of the ice‐crystals formed resulted in formation of a new cryogel with reduced pore‐sizes [51].

Presently, one of the most common strategies is affinity‐based separation processes. It is preferred to use porous support materials including monolithic ones such as macroporous cryogels in the separation process. In addition to their unique features of showing high porosity, mechanical stability, and durability, hydrophilic or hydrophobic characteristics in the formation of desired cryogels. Therefore, these properties make cryogels ideal for affinity separation [51].

In the separation process, a molecule with a specific recognition ability can be immobilized on cryogels. Target aimed to be separated by the complementary ligand into the solution mixture containing the target molecule under favourable conditions, and then, selectively recognized by passing it through a cryogel column. Afterwards, competitive ligand usage or application of changing physical parameters such as pH, ionic strength or temperature were performed. By the way, the interaction between ligand and target molecule is eliminated and the target molecules are obtained in a pure form. In the separation of a biomolecules, it is noteworthy to indicate that there are deterministic biological and physicochemical properties such as molecular size, net charge, bio‐specific properties and hydrophobicity. In this regard, immobilized metals, dyes, hydrophobic interactions and boronate‐based techniques are commonly applied according to the above‐mentioned properties of target molecule, and the summarized examples of studies performed with cryogels for the separation of molecules are reported in Table 3.

**TABLE 3:** The summarized examples of studies performed with cryogels for the separation of molecules

Polymer	Ligand	Target	Type of chromatography	Shape	Efficiency	Reusability (cycle)	Ref.
pHEMAT	1‐nNaphthylamine	Lysozyme	Hydrophobic affinity chromatography	Monolithic	105.8 mg/g	30	[52]
Epoxy activated polyacrylamide	Concanavalin A	*S. cerevisiae*	Affinity chromatography	Monolithic	93%	‐	[53]
poly(AAm‐MBAAm)	‐	Globulin	Steric exclusion chromatography	Monolithic	20 mg/mL	‐	[54]
pHEMA	Reactive Green HE 4B and Reactive Red 120	HIgG	Dye ligand affinity chromatography	Disc	Reactive Green HE 4B: 239.8 mg/g Reactive Red 120: 170 mg/g for RG functionalized CC	10	[55]
	Polyethyleneimine, polymyxin B and lysozyme	Endotoxin	Negative chromatography	Monolithic	Polyethyleneimine: %100 Polymyxin B: %91 Lysozyme: %100	‐	[56]
p(HEMA‐co‐GMA)	Sulfoalkylation	Lysozyme	Strong‐cation exchange chromatography	Monolithic	8 mg/mL	5	[57]
pHEMA	Cibacron Blue F3GA	Albumin	Dye ligand affinity chromatography	Monolithic	343 mg/g	10	[58]
pHEMA	Protein A	IgG	Affinity chromatography	Monolithic	83.2 mg/g	10	[59]
pHEMAH	MAH	plasmid DNA	Histidine affinity chromatography	Disc	13.5 mg/g	3	[60]

Abbreviations: GMA, glycidylmethacrylate; MAH, N‐methacryloyl‐(l)‐histidine methyl ester); MBAAM, methylen‐bis‐acrylamide; pAAM, poly(acrylamide); pHEMA, poly(2‐hydroxyethyl methacrylate; pHEMAH, poly(hydroxyethyl methacrylate‐N‐methacryloyl‐(l)‐histidine methyl ester); pHEMAT, poly(2‐hydroxyethyl methacrylate‐*co*‐*N*‐methacryloyl‐(L)‐tyrosine methyl ester).

Cryogels have low flow resistance, interconnected pores and reusability. Cryogels have many different applications aiming isolation of biomolecules. When dealing with biological samples containing even particulate materials including cells. Cryogels are of great interest on healthcare. They are robust and can be autoclaved for use on medical care. Besides, they can be used as scaffolds for tissue engineering. Cryogels were evaluated regarding clogging from microbial suspension/cell homogenates and other particulate‐rich media. It was demonstrated that bacterial cells could be separated by chromatography on cryogels.

Some limitations of cryogels were reported. Cryogels have lower surface area in comparison to that of other polymeric materials such as nano‐ and micron‐sized particles. These particles could be easily embedded into the cryogels to enhance surface area and novel composite cryogels were constructed. The overview of the composite cryogels applied for the separation of different targets is given in Table 4.

**TABLE 4:** The overview of the composite cryogels applied for the separation of different targets

Polymer	Composite material	Target	Type of chromatography	Sample	Efficiency	Ref.
PVA	Vinilpiridin particle	17β‐estradiol	Molecular imprinting technology	Aqueous solution	75.4% ± 0.6%	[61]
pHEMA	Glycidyl methacrylate beads	Albumin	Molecular imprinting technology	Human serum	16.3 mg/g	[62]
pHEMA	Cellulose beads	Lactoperoxidase	Cation exchange	Bovine whey	98.0%–99.8%	[63]
pAAM	Ethylene glycol dimethacrylate/N‐methacryloyl‐(l)‐histidine‐Cu(II) particle	Hyaluronic acid	Molecular imprinting technology	Fish eye and S. equi culture	318 mg/g	[64]
pHEMA	Cellulose beads	IgG and albümin	Anion‐exchange	Human serum	IgG: 83.2% lbümin: 98%	[65]
pHEMA	Poly(2‐hydroxyethyl methacrylate‐*co*‐*N*‐methacryloyl‐(L)‐tyrosine methyl ester) particles	Anti‐hepatitis B surface antibody	Molecular imprinting technology	Human plasma	701.4 mIU/g	[7]
pHEMA	Poly(2‐hydroxyethyl methacrylate‐N‐methacryloyl‐L‐histidine)‐Ni(II) microsphere	Hemoglobin	Molecular imprinting technology	Human blood	23.4 mg/g	[66]
pAAM	Fe_3_O_4_ nanoparticles	Albumin	Affinity chromatography	Aqueous solution	19‐20 mg/g	[67]
Cryogelation of molecularly imprinted nanoparticles	MAA based nano particle	Propranolol	Molecular imprinting technology	Aqueous solution and complex plasma sample	94%	[68]

Abbreviations: MAA, methacrylic acid; pAAM, poly(acrylamide); pHEMA, poly(2‐hydroxyethyl methacrylate; PVA, polyvinyl alcohol .

During the initial phase when evaluating the possible use of cryogels in biotechnology, then monomers based on acrylamide were used, but now some natural polymers also can be mixed into the system.

Polyethylenimine (PEI) has a branched structure. Increase surface area by lengthening polymer chains. Thus, it is used in the synthesis of suitable materials for adsorption. In addition, PEI can be used as an anion exchanger thanks to its amino groups. Wang and Sun prepared pHEMA‐based ion exchange composite cryogels to separate albumin. First, they prepared a poly(GMA‐EDMA) monolith, ground it and embedded it in p(HEMA) based cryogel. Then, the surface of the cryogels was modified with PEI and diethylaminoethyl (DEAE), both individually and in pairs, to give the cryogels anion‐exchange properties. The adsorption capacity was determined to be approximately 1.5–4.6 times higher than the cryogels obtained with single modifications [69].

Lim et al. prepared composite cryogels and coated the surface of PVA‐based cryogel beads with powdered activated carbon. They preferred PVA because it is non‐toxic to microorganisms. The aims were (1) removal of 4‐chlorophenol from wastewater and (2) ensuring its biodegradation [70].

When introducing stimuli‐responsive polymers in combination with cryogels it is possible to combine the best properties of each of the units. Temperature sensitive cryogels can show swelling property when temperature‐controlled water is added. The cryogel with stimuli‐responsive polymers may change properties in different direction [33]. It is obvious that the changes in conformation of smart polymers can be used to facilitate elution of captured substances from the polymer combination [42].

Cryogels and MIPs are combined when designing composites, then the affinity and stability properties of MIPs represents an advantage concerning affinity properties and stability. It was also demonstrated that building cryogels by crosslinking particles, either as latex particles, or even microbial cells is possible. This latter led to creation of a new type of microbial reactor since the microbial cells were alive and had high metabolic activity [71]. The large pores and the thin filaments led to efficient supply of nutrient to the cells and also removal of waste compounds.

**FIGURE 1:**
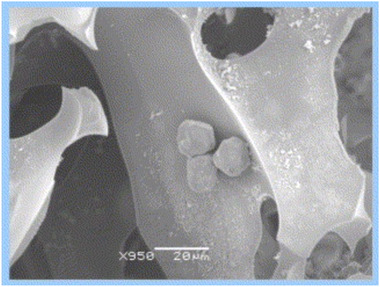
Scanning electron micrograph of the inner part of the supermacroporous cryogel‐protein A matrix loaded with lymphocytes. The cells are affinity bound on the pore walls. Magnification (× 950). Reproduced with permissions from [72]

In a study it was shown that one can separate mammalian cells from monolithic supermacroporous cryogels that were used for the specific fractionation and separation of human peripheral blood lymphocytes using a chromatographic approach. The affinity matrix was applied to generate a novel cell separation approach using the interaction of protein A with an affinity against IgG. Treatment of lymphocytes with goat anti‐human IgG(H+L) results in the effectively obtained IgG‐positive B‐lymphocytes from T‐lymphocytes. The mechanism is based on the interaction of protein A immobilized dimethyl acrylamide (DMAAm) monolithic cryogel matrix with IgG‐positive B‐lymphocytes. In this regard, unbound T‐lymphocytes passed through the column. Scanning electron microscope image of B‐lymphocytes captured on the cryogel matrix is indicated in Figure 1 [72].

Cryogel composites with MIPs led to interesting studies on treatment of wastewater. Chromatography of waste water is usually connected to low fluxes and frequent clogging problems. In one study Mattiasson et al. [73] addressed the problems with endocrine disruptors. Hormone‐like compounds are present at low concentrations, but they are very potent physiologically. The MIPs in the cryogel captured estrogen‐like compounds [73]. In order to make the cryogel composite adapted to the harsh conditions that is found in wastewater the gels were polymerized in a plastic housing which made it possible to use the gels under stirring while capturing impurities from the wastewater that was regarded as pure enough to be released to the recipient [74]. However, the MIPs which were produced in order to have receptors for capturing estrogen‐active compounds can also interact with estrogen receptors in living organisms. The efficiency of the MIPs was high since it was not possible to detect any residues in the treated water.

Similar studied were also made to evaluate the possibility to capture herbicides and other environmental pollutants. After removing the cryogel‐composite it was possible to release the captured material and then reuse the adsorbent.

A recent development is to combine cryogel production integrated with production of molecularly imprinting such that the affinity cavities will be present in the polymer filaments. Such structures seem to have high capacities for capturing pollutants.

Still another application was possible to carry out using the superporous cryogels. When dealing with phase display libraries, selection for the most relevant phages producing suitable ligands for the planned application is usually cumbersome. However, by immobilizing proteins on the cryogels, it was possible to first pass a library of phages which strongly bind to the immobilized protein. The surplus of phages were removed and the system was washed before microbial cells (*E. coli*) were introduced in order to infect the microbial cells with the phage carying the receptor for the target molecule [75]. The conventional sorting of the phages is called bio panning. It is time‐consuming and needs usually several repetitions in order to get a good result. The 'Chromato‐panning' when using cryogels turned out to be beneficial due to low time consumed and high resolution achieved [76].

An unexpected result obtained with highly porous (average pore size >X μm) cryogels is that mammalian cells can be stored in these large pores and such cryogels can be injected through a conventional syringe and a catheter. The gel is compressed while passing the catheter and the elasticity is sufficient to keep the cells viable. Such injection of cryogels are used in healthcare and reports so far are very promising [77].

Molecular imprinting method is based on the formation of a complex by interaction between a monomer which carries special functional group and a template molecule. Imprinted polymers having shape and size complementary along with chemical functionality were produced. It is possible to remove harmful or unwanted substances from the blood by molecular imprinting. High amount of cholesterol could be removed from the blood using molecularly imprinting and it works as a treatment method [78]. Cholesterol removal from the intestinal tract are mimicking solution by MIP technology is achieved by polymerization of the monomer, removal of the template molecules from the polymeric structure. The resulting free binding sites of the polymer are free for the recognition of selective molecules. In other words, the mechanism of molecular imprinting resembles the antigen‐antibody or enzyme‐substrate interaction principle. The recognition residues of the polymeric matrix interact with the target molecules and differentiate structurally.

Mosbach et al. introduced molecularly imprinting. As mentioned above, MIPs have potential to be used in many applications [71]. Furthermore, MIPs are far more stable than biomolecules and one can then reuse them repeated times [72]. The overview of the molecularly imprinted cryogels applied for the separation of different targets is given in Table 5.

**TABLE 5:** The overview of the molecularly imprinted cryogels applied for the separation of different targets

Polymer	Target	Desorption agent	Shape	Efficiency	Sample	Analytical methods	Ref.
Poly(2‐hydroxyethyl methacrylate)‐*co*‐*N*‐methacryloyl‐(l)‐glutamic acid‐Fe^3+^	L‐glutamic acid	100 mM HNO_3_	Monolithic	11.34 μmol/g	Aqueous solution	HPLC	[79]
Poly(2‐hydroxyethyl methacrylate)‐*co*‐*N*‐methacryloyl‐(l)‐ phenylalanine	*l* ‐phenylalanine	%50 (v/v) ethyleneglycol/water	membrane	24.1 mg/g	Aqueous solution	FPLC	[80]
Poly(2‐hydroxyethyl methacrylate‐*co*‐*N*‐methacryloyl‐(l)‐cysteine methyl ester	Fe^3+^	0.1 M EDTA	Monolithic	75 μg/g	Human plasma	AAS	[81]
Poly(2‐hydroxyethyl methacrylate) and *N*‐methacryloyl‐l‐histidine methylester‐Cu^2+^	Protein C	1.0 M NaCl	Monolithic	30.4 mg/g	Aqueous solution	FPLC	[82]
Poly(2‐hydroxyethyl methacrylate)‐*co*‐(*N*‐methacryloyl‐l‐histidine methyl ester)	Hemoglobin	Acetate buffer containing 10% ethylene glycol (0.1 m, pH 4.0)	Monolithic	167.4 mg/g	Human blood	HPLC	[83]
Poly(2‐hydroxyethyl methacrylate)‐co‐N‐methacryloylamidoantipyrine‐Ce(III)	Myoglobin	0.1 M NaOH‐Na_2_SO_4_	Monolithic	68 mg/g	Human serum	UV‐VIS‐Near Infrared spectroscopy	[84]
Poly (2‐hydroxyethyl methacrylate)‐N‐methacryloyl‐(L)‐histidin‐Cu(II)	N‐Acetylneuraminic acid	0.1 M Na_2_CO_3_‐NaOH	Monolithic	83.2 mg/g	Human serum	UV‐VIS‐Near Infrared spectroscopy	[85]
Poly(2‐hydroxyethyl methacrylate) and N‐methacryloylamido antipyrine	Cerium(III)	23.4 mol/L HCl	Monolithic	36.58 mg/g	Aqueous solutions and bastnäsite ore samples	ICP‐MS	[86]
Poly(2 hydroxyethyl methacrylate)‐*co*‐(*N*‐methacryloyl‐l‐histidine methyl ester)	Concanavalin A	0.1 M phosphate buffer containing 1.0 mM MnCI_2_, 1.0 mM MgCl_2_, 1.0 mM CaCl_2_ and 1.0 M NaCl (pH 7.0)	Monolithic	4.91 mg/g	Jack bean extract	FPLC	[87]

Abbreviations: AAS, atomic absorption spectrometry; FPLC, fast protein liquid chromatography; HPLC, high performance liquid chromatography; ICP‐MS, inductively coupled plasma mass spectrometry.

This technology includes the usage of synthetic polymers for the target [88]. This promising approach is nowadays widespread and MIPs could be commonly applied for separation and sensing [89]. Combining MIPs with biosensors creates interesting possibilities. We have used the combined technologies successfully. A global trend comprises miniaturization of the MIPs and the biosensors [90]. Affinity separation by molecular imprinting is cheaper than other methods. Besides, MIPs are more stable and more preferable because they are used for a longer time than that of other competitive ones.

In the case of combining production of cryogels and MIPs. The strategy to combine these two processes was to mix the monomer solution and its catalysts with a solution containing the target molecule which should be used to form the cavity in the molecularly imprinted polymer; that means that the thin polymer filaments in the cryogel will all have affinity cavities all through the cryogel. In the case that MIPs are produced in presence of stimuli‐responsive polymers one can expect similar reactions as were presented for the cryogels. In a previous study, Denizli and co‐workers designed IgG imprinted thermosensitive NIPAm based monolithic cryogel for the purification of IgG. NIPAm was preferred as thermo‐responsive monomer and maximum IgG binding capacity of the proposed cryogel was determined as 106.1 mg/g, obtained at pH 7.4 and 40 °C. Selectivity and applicability of the imprinted cryogel from human plasma was verified. Consequently, macroporous structure of cryogel enables IgG purification with the assistance of the thermos responsive property of NIPAm providing controlled binding‐elution advantage by changing temperature.

## 4 CONCLUSION

It has been clearly emphasized above that there are many methods applied for bio‐separation and their application areas. Well‐tested methods with new techniques such as combination of the processes offered. The complexity of the problems in bio‐separation area leads to the emergence of some challenges. To meet the requirements, interdisciplinary attempts were made and presently a new field of separation needs novel engineered steps.

## CONFLICT OF INTEREST

The authors have declared no conflict of interest.
